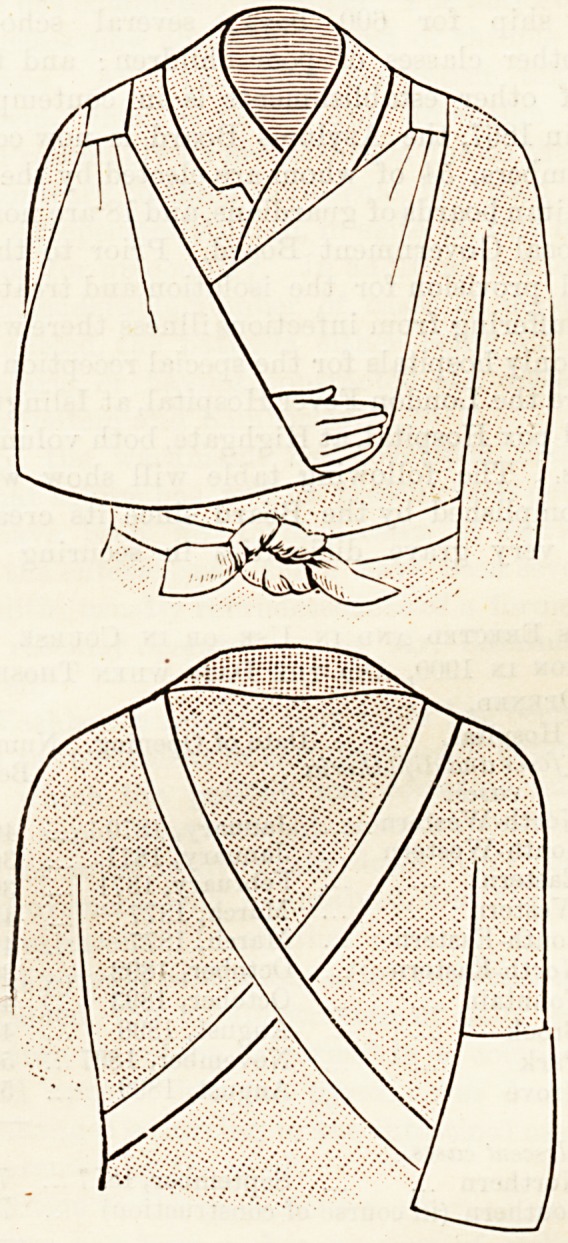# New Appliances and Things Medical

**Published:** 1900-09-22

**Authors:** 


### NEW APPLIANCES AND THINGS MEDICAL.

**[We shall be glad to receive, at our Office, 28 & 29, Southampton Street, Strand, London, W.C., from the manufacturers, specimens of all new preparations and appliances which may be brought out fromti me to time.]**

### MERCUROL.

(Parke, Davis, and Co., 111, Queen Victoria Street, London, E.C.)

Mercurol is a new compound of mercury with nuclein prepared from yeast. It has the valuable properties of being soluble in water and of not coagulating albumen. The combination of these two properties in a metallic compound with the high antiseptic powers of mercury renders the preparation one of extreme value in dermatological and surgical practice. It is claimed for this new germicide that it has a selective antagonism to the gonococcus, and hence for syringing the urethra, when infected with this specific microbe, the best results possible from local antiseptic treatment may be reasonably expected. In addition to the valuable properties already mentioned, mercurol is neither caustic nor corrosive, and for this reason it may be boldly applied to delicate and tender mucous membranes, the seats of microbic infection; and especially in cases of ophthalmia neonatorum will these advantages be particularly appreciated. Solutions of mercurol are not permanent, and for this reason the drug should be kept in the powder form, and dissolved in water as occasion may require. For practical purposes the small capsule supplied with each bottle should be filled with the powder and shaken into a six-ounce bottle containing four ounces of distilled water. Slight agitation will effect complete solution, and a 1 per cent. mercurol lotion, which is sufficiently strong for ordinary purposes, will result.

### NEW ARM SLING.

(Sanitary Wood-Wool Company, Limited, 26, Thavie's Inn, Holborn Circus, London, E.C.)

The new patent arm sling—the illustration of which we append—is a simple yet ingenious contrivance for obviating the pressure which is necessarily exercised at the back of the neck when the ordinary sling is employed with the knot behind, or on the shoulder. As an examination of the illustrations will at once demonstrate, the sling is held in position by bands which pass over the shoulders, and are crossed as they are carried over the back and round the waist, the ends being fastened by a bow in front. The pressure is thus equably distributed, and the weight of the suspended arm borne by the strong muscles of the back and trunk: the annoyance and discomfort of a knot at the nape of the neck or elsewhere is thus avoided. The contrivance is very simple, and, inasmuch as the sling can be turned inside out, it can be indifferently applied for the support of either right or left arm. For injuries of the upper extremities it should be a particularly useful and handy bandage; for fractures in plaster of Paris or adjustable splints the ease with which it can be fitted should appeal to surgeons and casualty-room officers.

## Figures and Tables

**Figure f1:**